# Equivalent Circuit to Analyze the Transmitting Characteristics of a Cymbal Array

**DOI:** 10.3390/s22228743

**Published:** 2022-11-12

**Authors:** Hayeong Shim, Kyungseop Kim, Heeseon Seo, Yongrae Roh

**Affiliations:** 1 School of Mechanical Engineering, Kyungpook National University, Daegu 41566, Korea; 2 Agency for Defense Development, Changwon 51678, Korea

**Keywords:** cymbal transducer, equivalent circuit, broadband transducer, cymbal array

## Abstract

A cymbal transducer has a simple structure consisting of a piezoceramic disk and metallic caps and has broadband characteristics when built as an array. The finite element method (FEM) is generally used to analyze the characteristics of acoustic transducers. However, the FEM requires a longer analysis time as the model becomes larger, which makes it limited and less efficient for analyzing the cymbal array. In this study, a new equivalent circuit with higher efficiency and accuracy, comparable to that of the FEM, was proposed to analyze the performance of cymbal arrays. The equivalent circuit for the array was constructed by connecting the equivalent circuits of individual cymbal transducers in parallel with a radiation impedance matrix that included both the self- and mutual radiation characteristics of the array. The validity of the new equivalent circuit was verified by measuring the transmitting voltage response of a cymbal array specimen and comparing it with that calculated using the circuit. The comparison confirmed the efficiency of the equivalent circuit in analyzing the characteristics of the cymbal array. The proposed equivalent circuit can facilitate the design of a large array of cymbal transducers.

## 1. Introduction

A cymbal transducer is a miniaturized version of the class V flextensional transducer [1]. It consists of a piezoceramic disk and a pair of metal caps and has a low resonant frequency considering its small size. Broadband characteristics can be obtained when cymbals are arranged and used as an array [2].

Since the development of the cymbal transducer by Newnham et al. in the 1990s, various studies have been conducted by many researchers [3,4,5,6,7,8]. The performance of cymbal transducers has generally been analyzed using the finite element method (FEM). However, an equivalent circuit for cymbal transducers has recently been developed [9,10]. However, in the case of the cymbal array, an equivalent circuit for exclusive use has not yet been developed, so the FEM or experimental analysis methods are still mainly used. For instance, Tressler et al. experimentally analyzed and compared the transmitting voltage response (TVR) of a cymbal array for different constituent materials and construction methods [11]. Zhang et al. analyzed the displacement of each element constituting a cymbal array and calculated the TVR while changing the spacing between the elements using the FEM [12]. Rajapan et al. fabricated cymbal array specimens with various encapsulation materials and measured their acoustic properties [13]. Du et al. analyzed the acoustic properties of a cymbal array using an experimental method [14]. Kim et al. derived an optimized array structure to maximize the bandwidth of a cymbal array using the FEM [15].

The analysis of the acoustic characteristics of the cymbal array as conducted in all these studies using the FEM or the experimental method is very time-consuming. The cymbal transducer is small compared to the wavelength at the resonant frequency. This small and curved geometry of the cymbal requires elaborate modeling and meshing, which prolongs the analysis time so much that using the FEM to analyze a cymbal array larger than a 3 × 3 array would hardly ever be feasible.

There are positive indications that the difficulty of analyzing the cymbal array using the FEM can be resolved by developing a dedicated equivalent circuit, as inspired by the study conducted by Pyo and Roh [16]. Several related studies have been conducted. As a case in point, Zhang et al. analyzed the cymbal array using the equivalent circuit method (ECM). However, the discrepancy between the measured and analyzed TVR spectra was not ignorable. This was attributed to the inaccuracy in the mutual radiation impedance between the cymbals in the array [17]. It is necessary to supplement the equivalent circuit by deriving an accurate radiation impedance matrix dedicated to the cymbal array.

In the case of cymbal arrays, the mutual radiation impedance has a significantly large impact on the acoustic characteristics of the array because a large number of transducers, smaller than the wavelength, are arranged within a narrow area [18]. A typical equivalent circuit does not reflect such mutual radiation impedance but considers only the self-radiation impedance of each transducer [19]. The mutual radiation impedance in an array has been studied in several studies using an equivalent circuit [20,21]. Oguz et al. analyzed the effect of mutual radiation impedance on each cell in a capacitive micromachined ultrasonic transducer array using an equivalent circuit [22]. Akhbari et al. developed equivalent circuits for arrays of curved and flat piezoelectric micromachined ultrasonic transducers and analyzed the radiation characteristics of arrays using the circuits [23]. Pyo and Roh designed a structure that maximized the bandwidth of a planar Tonpilz array using an equivalent circuit that included the mutual radiation impedance [16]. Sim et al. developed an equivalent circuit model that could predict the acoustic response of a cylindrical Tonpilz array by considering the mutual radiation impedance [24]. However, all these studies were related to arrays composed of transducers with a single radiating surface. Because the upper and lower caps of a cymbal transducer act as independent radiating surfaces, as noted in Zhang’s work [17], the radiation impedance matrix used in previous studies is unsuitable for a cymbal array. The dual piston model in [17] was observed to be insufficient to express the interaction between elements in a cymbal array because the comparison between the measured and calculated results showed considerable differences.

This study proposes a new equivalent circuit that can accurately and efficiently resolve this problem and analyze the acoustic characteristics of a cymbal array. We developed a new radiation impedance matrix that could accurately reflect the interaction between cymbal transducers’ dual radiating surfaces and combined this matrix with the equivalent circuits of individual cymbal transducers to analyze the characteristics of the cymbal array. The validity of the whole circuit was verified by comparing the analyzed TVR spectrum with that obtained from measurements with an experimental cymbal array specimen. The efficiency of the developed circuit in terms of the analysis time is discussed in comparison with the FEM.

## 2. Equivalent Circuit of a Cymbal Array

**Figure 1:**
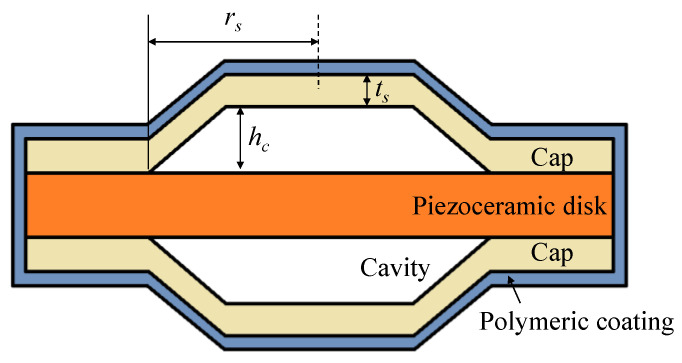
Schematic showing the structure of the cymbal transducer.

**Figure 2:**
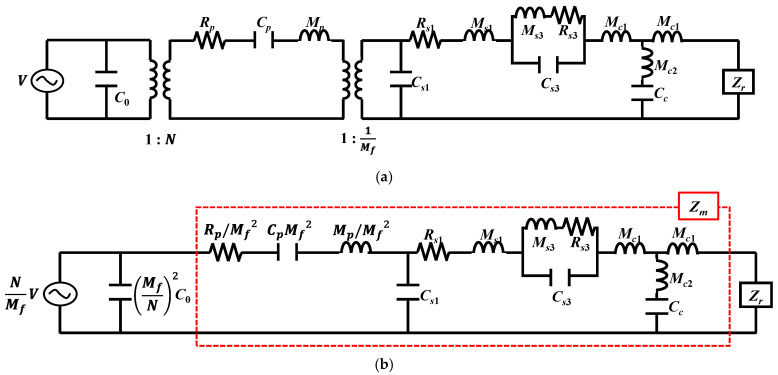
Equivalent circuit of the cymbal transducer: (**a**) with transformers, (**b**) transformed circuit.

The equivalent circuit of the cymbal array was composed by connecting the equivalent circuits of individual cymbal transducers in parallel and supplementing the interaction between the cymbal transducers with an acoustic impedance network. The structure of the cymbal transducer considered in this study is shown in Figure 1, where the structural parameters $t_{s}$, $h_{c}$, and $r_{s}$ are the thickness, cavity height, and base radius of the cap, respectively. Characteristics of the cymbal transducer are represented by the equivalent circuit, as shown in Figure 2, which duly incorporates and reflects both the fundamental and third harmonic mode vibrations of the transducer [10].

**Figure 3:**
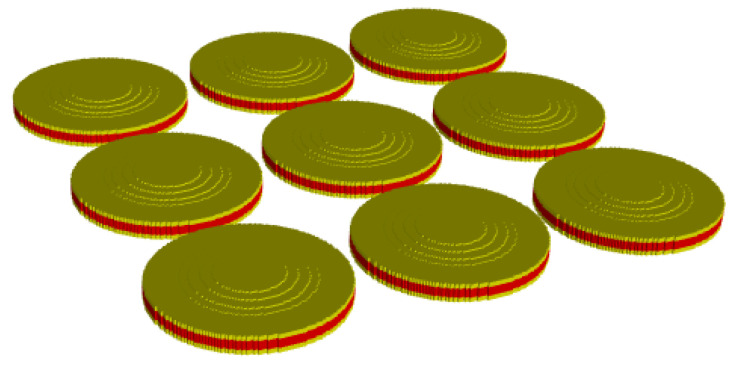
The cymbal array.

**Figure 4:**
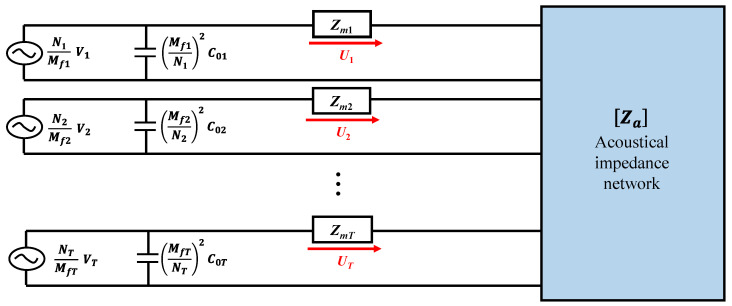
Equivalent circuit of the cymbal array.

In Figure 2, *V* is the input voltage, while $C_{0}$ and N are the clamped electrostatic capacitance and radial mode turns ratio of the piezoceramic disk, respectively. $R_{p}$, $C_{p}$, and $M_{p}$ are the mechanical impedances of the piezoceramic disk, and $M_{f}$ is the amplification factor of the cap. $C_{s1}$, $M_{s1}$, and $R_{s1}$ are the mechanical impedances related to the first vibration mode, whereas $C_{s3}$, $M_{s3}$, and $R_{s3}$ are those related to the third vibration mode. $M_{c1}$, $M_{c2}$, and $C_{c}$ are the mechanical impedances of the polymeric coating, whereas $Z_{r}$ is the radiation impedance. To build the circuit for the array, the transformers in the individual cymbal transducer circuit in Figure 2a were removed by transferring both the electrical voltage source and piezoceramic branches to the mechanical branch of the transducer, as shown in Figure 2b. In Figure 2b, $Z_{m}$ is the sum of all impedance parameters except $C_{0}$ and $Z_{r}$. The circuits for all the cymbal transducers constituting the array in Figure 3 were connected in parallel to form the combined equivalent circuit of the cymbal array, as shown in Figure 4.

In Figure 4, the subscript T is the total number of cymbals in the array, while $V_{i}$, $N_{i}$, $M_{fi}$, $C_{0i}$, $Z_{mi}$, and $U_{i}$ are the input voltage, piezoelectric turns ratio, amplification factor of the cap, clamped capacitance of the piezoceramic disk, mechanical impedance, and volumetric velocity of the *i*th cymbal, respectively. $\left[Z_{a}\right]$ is the acoustical impedance network matrix composed of the self-radiation impedance of the cymbals constituting the array and mutual radiation impedance between the cymbals [16]. Unlike previous studies, a new form of the acoustic impedance network was developed in this study to incorporate the exact mutual radiation impedance for a more accurate analysis of the acoustic characteristics of the cymbal array.

### 2.1. Mutual Radiation Impedance

**Figure 5:**
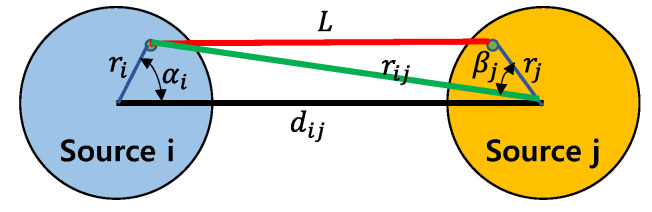
Arrangement of two piston sources.

Figure 5 shows two single-mode piston sources, i and j, with radii, a and b, respectively, and are separated by a distance, $d_{ij}$. The mutual radiation impedance, $Z_{ij}$, between two sound sources, is frequently calculated using Equation (1) [25]:

(1) $$Z_{ij}=2\rho_{r}c_{r}\pi a^{2}\overset{\infty }{\underset{s=0}{\sum }}\frac{1}{\sqrt{\pi }}\Gamma \left(s+\frac{1}{2}\right){\left(\frac{a}{d_{ij}}\right)}^{p}h_{p}^{\left(2\right)}\left(k_{r}d_{ij}\right)\times \overset{s}{\underset{n=0}{\sum }}{\left(\frac{b}{a}\right)}^{n+1}\left\{\frac{J_{s-n+1}\left(k_{r}a\right)J_{n+1}\left(k_{r}b\right)}{n!\left(s-n\right)!}\right\},$$

where $\rho_{r}$ and $c_{r}$ are the density and sound speed of the radiation medium, respectively, and $k_{r}$ is the wave number. Γ is the gamma function; $h_{p}^{\left(2\right)}$ is the spherical Hankel function; and J is the Bessel function of the first kind. Several studies have used this equation to express the mutual radiation impedance between sound sources of a single vibration mode. However, the equivalent circuit of the cymbal transducer used in this study represents both the first and third mode vibrations of the cap [10]. Hence, using Equation (1) when deriving the acoustic impedance network for the multimode cymbal array is likely to decrease the accuracy of the analysis. Hence, a new $Z_{ij}$ was derived in this study by incorporating the multimode vibration of the cymbal transducer.

The general form of the mutual radiation impedance between the two sound sources in Figure 5 is expressed as Equation (2) [25]. In Figure 5, $L^{2}=r_{ij}^{2}+r_{j}^{2}-2r_{j}r_{ij}\cos\left(\beta_{j}\right)$; $r_{ij}^{2}=d_{ij}^{2}+r_{i}^{2}-2r_{i}d_{ij}\cos\left(\alpha_{i}\right)$, where $r_{i}$ and $r_{j}$ are the distances from the designated point on sources i and j to the center of the sources, respectively. $\alpha_{i}$ is the angle between the horizontal axis and line, ${\vec{r}}_{i}$. $\beta_{j}$ is the angle between line ${\vec{r}}_{j}$ and the line connecting the designated point on source i and the center of source j. In Equation (2), $p(r_{ij})$ is the sound pressure generated by source i to a point on source j; $u(r_{i})$ is the velocity of source i; $u(r_{j})$ is the velocity of source j; and $u^{*}(r_{i})$ is the complex conjugate of $u(r_{i})$ [25].

(2) $$Z_{ij}=\frac{1}{\left|u_{a}\right|\left|u_{b}\right|}\int_{0}^{2\pi }\int_{0}^{a}p\left(r_{ij}\right)u^{*}\left(r_{i}\right)r_{i}dr_{i}d\alpha_{i},$$

where

$$u_{a}=\frac{1}{\pi a^{2}}\int_{0}^{2\pi }\int_{0}^{a}u\left(r_{i}\right)r_{i}dr_{i}d\alpha_{i},$$

$$u_{b}=\frac{1}{\pi b^{2}}\int_{0}^{2\pi }\int_{0}^{b}u\left(r_{j}\right)r_{j}dr_{j}d\beta_{j},$$

$$p\left(r_{ij}\right)=\frac{i\rho_{r}c_{r}k}{2\pi }\int_{0}^{2\pi }\int_{0}^{b}\frac{u\left(r_{j}\right)}{L}e^{-ikR}r_{j}dr_{j}d\beta_{j}$$

In [10], the velocity of the cymbal cap was derived using Equation (3), which includes both the first and the third mode vibrations. By substituting Equation (3) into Equation (2), the mutual radiation impedance of the multimode cymbal transducer can be calculated.

(3) $$u\left(r,t\right)=i\omega_{r1}\left[C_{11}J_{0}\left(\lambda_{1}r\right)+C_{21}I_{0}\left(\lambda_{1}r\right)+C_{31}\right]e^{i\omega_{r1}t}+i\omega_{r3}\frac{A_{3}}{A_{1}}\left[C_{13}J_{0}\left(\lambda_{3}r\right)+C_{23}I_{0}\left(\lambda_{3}r\right)+C_{33}\right]e^{i\omega_{r3}t},$$

where

$\omega_{r1}$, $\omega_{r3}$ = angular natural frequencies of the cap;

$A_{1}$, $A_{3}$ = modal participation factors of the first and third modes, respectively;

$\lambda_{1}^{4}=\frac{\rho_{s}t_{s}}{D}\omega_{r1}^{2}-\frac{t_{s}Y_{s}}{\Lambda^{2}D}$;

$\lambda_{3}^{4}=\frac{\rho_{s}t_{s}}{D}\omega_{r3}^{2}-\frac{t_{s}Y_{s}}{\Lambda^{2}D}$; 

$D=\frac{Y_{s}t_{s}{}^{2}}{12\;\left(1-\nu_{s}{\;}^{2}\right)}$; 

$\Lambda =\frac{r_{s}{}^{2}+{\left(h_{c}+\frac{t_{s}}{2}\right)}^{2}}{2}$; 

$C_{11}$, $C_{21}$, $C_{31}$, $C_{13}$, $C_{23}$, $C_{33}$ = constants;

$J_{0}$ = Bessel function of the first kind of order zero; 

$I_{0}$ = modified Bessel function of the first kind of order zero; 

$Y_{s}$ = Young’s modulus of the cap material;

$\nu_{s}$ = Poisson’s ratio of the cap material;

$\rho_{s}$ = density of the cap material.

**Figure 6:**
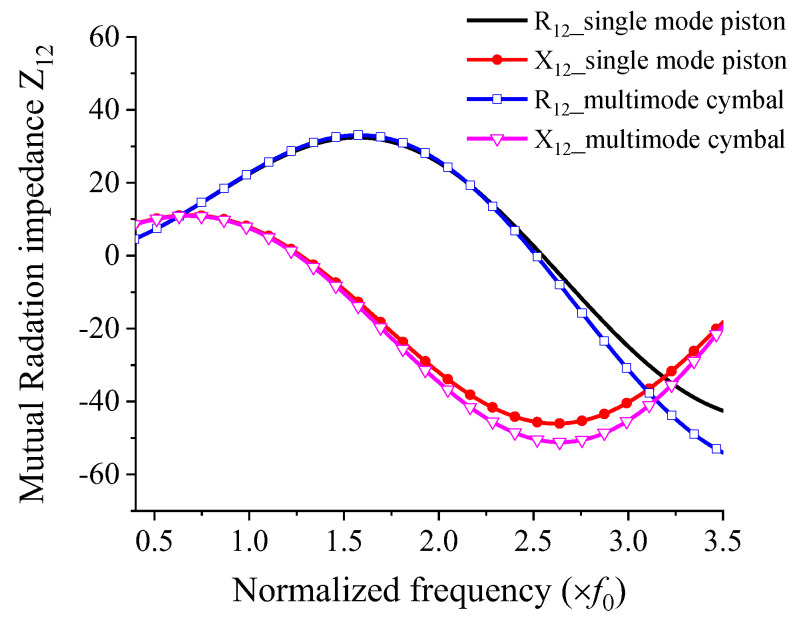
Comparison of the mutual radiation impedances of a single–mode piston source and muti–mode cymbal transducer; R_12_ = resistance, X_12_ = reactance.

The $t_{s}$, $h_{c}$, and $r_{s}$ are shown in Figure 1. Figure 6 shows the mutual radiation impedance when two cymbal transducers are arranged side-by-side, as shown in Figure 5. The curves of the single-mode piston were calculated using Equation (1), and those of the multimode cymbal were calculated using Equation (2). In the graph, R_12_ is the mutual radiation resistance, and X_12_ is the mutual radiation reactance between the two cymbal transducers. All frequencies were normalized to $f_{0}$, where $f_{0}$ is the frequency at which the cymbal transducer has its peak TVR level, as presented in Section 3. In Figure 6, the mutual radiation impedances of the single-mode piston source and multimode cymbal are almost identical in the low-frequency region. However, the difference between the two sets of impedances increases at higher frequencies. This comparison confirmed that the mutual radiation impedance derived in this study could accurately reflect the multimode vibration of the cymbal.

### 2.2. Acoustic Impedance Network

**Figure 7:**
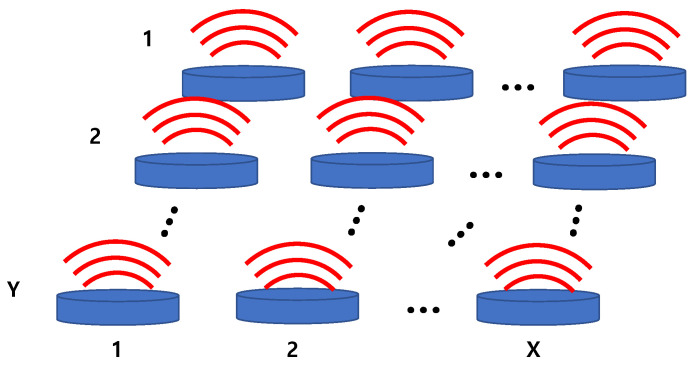
Sound radiation by an X × Y array of transducers with a single radiation surface.

The acoustic impedance network shown in Figure 4 is a matrix composed of self- and mutual radiation impedances. As shown in Figure 7, the acoustic impedance network $\left[Z_{a}\right]$ of the array composed of T transducers with a single radiation surface can be expressed as a T × T matrix, as shown in Equation (4), where X × Y = T [26]. Diagonal elements of the matrix represent the self-radiation impedance of each transducer, while the non-diagonal elements represent the mutual radiation impedance between the transducers.

(4) $$\left[Z_{a}\right]=\left[\begin{array}{cccc} Z_{11} & Z_{12} & \cdots & Z_{1T}\\ Z_{21} & Z_{22} & \cdots & Z_{2T}\\ \vdots & \vdots & \ddots & \vdots \\ Z_{T1} & Z_{T2} & \cdots & Z_{TT} \end{array}\right]$$

Typical transducers, such as Tonpilz, have a single radiation surface. In contrast, a cymbal transducer has upper and lower caps, each of which acts as a separate radiation surface. Therefore, the conventional acoustic impedance network in Equation (4) may not accurately represent the interactions between cymbals constituting the array. Hence, Zhang et al. calculated the radiation impedance of a cymbal array by considering each cymbal transducer as a dual-piston source [17]. However, there was a significant difference between the TVR spectra from the measurement and the dual-piston source analysis of the cymbal array. Therefore, in this study, we developed a new acoustic impedance network for a cymbal array by supplementing the dual-source concept with a more rigorous representation of the interaction between the upper and lower caps of the cymbal transducers in an array.

**Figure 8:**
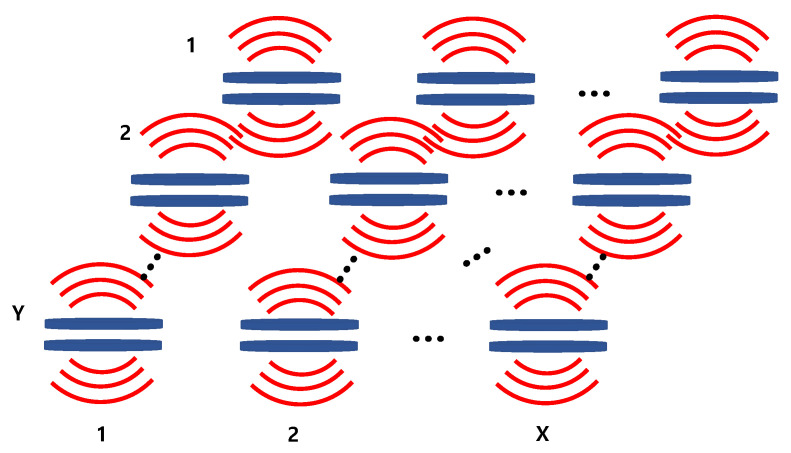
Sound radiation by an X × Y array of cymbal transducers with dual radiation surfaces.

If a cymbal transducer is regarded as a combination of two sources corresponding to the upper and lower caps, the cymbal array can be represented as illustrated in Figure 8, where the upper caps of the cymbals constitute the top surface, and the lower caps constitute the bottom surface.

If the total number of cymbal transducers constituting the array is T, the dual-source array can be regarded as consisting of 2T sources. The mutual radiation impedance ${\left[Z_{a}\right]}_{m,top}$ between the sound sources located at the top surface of the array can be expressed as Equation (5). Each element of the impedance matrix is obtained using Equation (2).

(5) $${\left[Z_{a}\right]}_{m,top}=\left[\begin{array}{cccc} 0 & Z_{12} & \cdots & Z_{1T}\\ Z_{21} & 0 & \cdots & Z_{2T}\\ \vdots & \vdots & \ddots & \vdots \\ Z_{T1} & Z_{T2} & \cdots & 0 \end{array}\right]$$

Similarly, the mutual radiation impedance, ${\left[Z_{a}\right]}_{m,bottom}$, between the sources located at the bottom surface of the array can be expressed as Equation (6).

(6) $${\left[Z_{a}\right]}_{m,bottom}=\left[\begin{array}{cccc} 0 & Z_{T+1\;T+2} & \cdots & Z_{T+1\;2T}\\ Z_{T+2\;T+1} & 0 & \cdots & Z_{T+2\;2T}\\ \vdots & \vdots & \ddots & \vdots \\ Z_{2T\;T+1} & Z_{2T\;T+2} & \cdots & 0 \end{array}\right]$$

Equations (5) and (6) only consider the interaction between the sound sources located on the same surface, assuming that there is a baffle preventing interaction between the sources on the top and bottom surfaces. In an actual array, the upper and lower caps of the same cymbal are disconnected by a piezoceramic disk. However, the upper cap of a cymbal cannot be seen as completely disconnected from the lower cap of a neighboring cymbal. Therefore, the interaction between the upper caps on the top surface and neighboring lower caps on the bottom surface of the cymbal array is expressed as Equation (7).

(7) $${\left[Z_{a}\right]}_{m,cross}=\left[\begin{array}{cccc} 0 & Z_{T+1\;2} & \cdots & Z_{T+1\;T}\\ Z_{T+2\;1} & 0 & \cdots & Z_{T+2\;T}\\ \vdots & \vdots & \ddots & \vdots \\ Z_{2T\;1} & Z_{2T\;2} & \cdots & 0 \end{array}\right]$$

The total mutual radiation impedance, ${\left[Z_{a}\right]}_{m}$, of a cymbal array radiating in the direction normal to the top surface is expressed as Equation (8), by adding all mutual radiation impedance matrices in Equations (5)–(7). As there are T cymbals in the array, the total mutual radiation impedance matrix is a T × T matrix.

(8) $$\begin{array}{l} {\left[Z_{a}\right]}_{m}={\left[Z_{a}\right]}_{m,top}+{\left[Z_{a}\right]}_{m,bottom}+{\left[Z_{a}\right]}_{m,cross}\\ =\left[\begin{array}{cccc} 0 & Z_{12}+Z_{T+1\;T+2}+Z_{T+1\;2} & \cdots & Z_{1T}+Z_{T+1\;2T}+Z_{T+1\;T}\\ Z_{21}+Z_{T+2\;T+1}+Z_{T+2\;1} & 0 & \cdots & Z_{2T}+Z_{T+2\;2T}+Z_{T+2\;T}\\ \vdots & \vdots & \ddots & \vdots \\ Z_{T1}+Z_{2T\;T+1}+Z_{2T\;1} & Z_{2T\;2} & \cdots & 0 \end{array}\right] \end{array}$$

The self-radiation impedance ${\left[Z_{a}\right]}_{s}$ of the cymbal array is expressed as Equation (9) [26].

(9) $${\left[Z_{a}\right]}_{s}=\left[\begin{array}{cccc} Z_{11} & 0 & \cdots & 0\\ 0 & Z_{22} & \cdots & 0\\ 0 & 0 & \ddots & \vdots \\ 0 & 0 & \cdots & Z_{TT} \end{array}\right]$$

The final acoustic impedance network, $\left[Z_{a}\right]$, of a cymbal array is obtained by adding Equations (8) and (9) as a combination of self- and mutual-radiation impedances, as shown in Equation (10) [26].

(10) $$\left[Z_{a}\right]={\left[Z_{a}\right]}_{s}+{\left[Z_{a}\right]}_{m}=\left[\begin{array}{cccc} Z_{11} & Z_{12}+Z_{T+1\;T+2}+Z_{T+1\;2} & \cdots & Z_{1T}+Z_{T+1\;2T}+Z_{T+1\;T}\\ Z_{21}+Z_{T+2\;T+1}+Z_{T+2\;1} & Z_{22} & \cdots & Z_{2T}+Z_{T+2\;2T}+Z_{T+2\;T}\\ \vdots & \vdots & \ddots & \vdots \\ Z_{T1}+Z_{2T\;T+1}+Z_{2T\;1} & Z_{2T\;2} & \cdots & Z_{TT} \end{array}\right]$$

### 2.3. Sound Pressure from the Array

**Figure 9:**
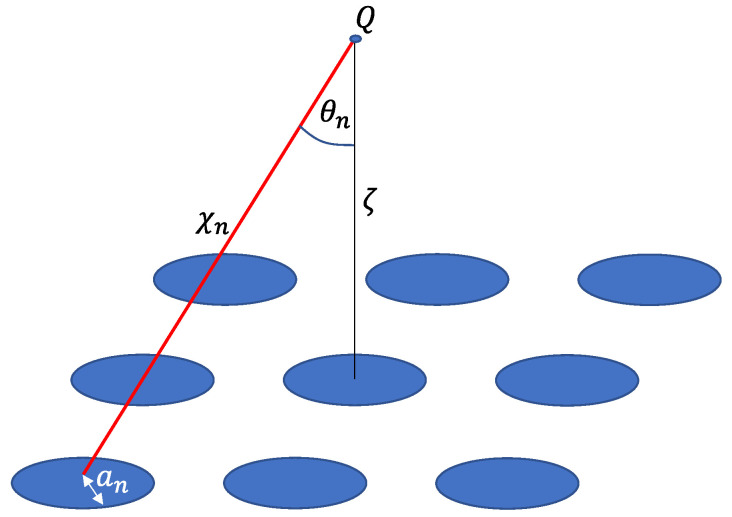
Geometry of the planar cymbal array.

The sound pressure radiated from the cymbal array can be calculated by combining the equivalent circuit of the cymbal array in Figure 4 and the acoustic impedance network derived in the previous section. The mechanical impedance, [$Z_{m}$], of the T-cymbal transducers constituting the array can be represented by the matrix in Equation (11) [26]. The voltage applied to each transducer is expressed as Equation (12) [26]. Combining these two equations with the acoustic impedance in Equation (10), the volumetric velocity, [*U*], of the cymbal array can be calculated using Equation (13) [23]. By substituting [*U*] from Equation (13) into Equation (14), we can determine the sound pressure, p, measured at point Q at a vertical distance, ζ, from the center of the array, as shown in Figure 9 [25].

(11) $$\left[Z_{a}\right]=\left[\begin{array}{cccc} Z_{m1} & 0 & 0 & 0\\ 0 & Z_{m2} & 0 & 0\\ 0 & 0 & \ddots & 0\\ 0 & 0 & 0 & Z_{mT} \end{array}\right],$$

(12) $$\left[V\right]=\left[\begin{array}{c} \begin{array}{c} \frac{N1}{M_{f1}}V_{1}\\ \frac{N_{2}}{M_{f2}}V_{2} \end{array}\\ \begin{array}{c} \vdots \\ \frac{N_{T}}{M_{fT}}V_{T} \end{array} \end{array}\right],$$

(13) $$\left[U\right]={\left(\left[Z_{m}\right]+\left[Z_{a}\right]\right)}^{-1}\times \left[V\right],$$

(14) $$p\left(\zeta \right)=\overset{T}{\underset{n=1}{\sum }}\left[i\frac{\rho_{r}c_{r}k_{r}a_{n}^{2}U_{n}}{2\chi_{n}}\times \Theta \left(\theta_{n}\right)e^{i\left(\omega t-k_{r}\chi_{n}\right)}\right],$$

where ω is the angular frequency; t is the time; $\chi_{n}$ is the distance between the nth cymbal and the measurement point Q; $\theta_{n}$ is the angle between the line connecting point Q to the nth cymbal and the vertical axis passing through the array center; and Θ is the directional factor of each cymbal. Using the acoustic pressure obtained from Equation (14), the TVR of the cymbal array can be calculated, and the radiation characteristics of the array can subsequently be analyzed.

## 3. Validation of the Equivalent Circuit Analysis

**Figure 10:**
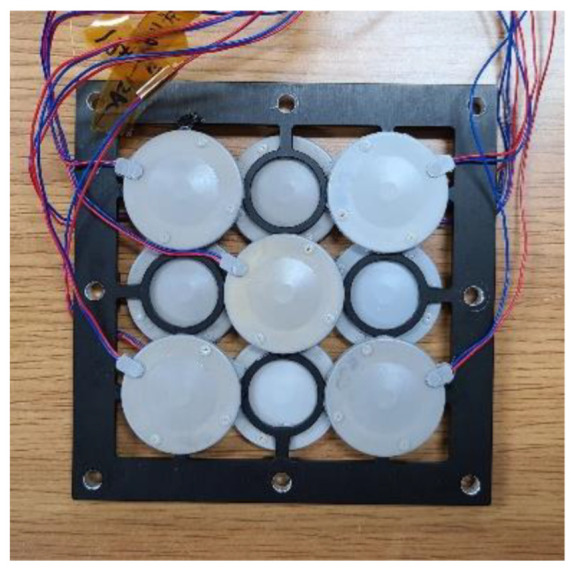
Photograph of the cymbal array specimen.

To validate the analysis, we fabricated and evaluated the characteristics of a 3 × 3 cymbal array specimen, as shown in Figure 10. The array had a dual-layer structure, and the horizontal center-to-center spacing between cymbals in the array was 23.6 mm [27]. Although an aluminum frame was used to fix the cymbal transducers, the frame did not affect the acoustic characteristics of the array; therefore, it was not considered in the equivalent circuit. The piezoceramic used was PZT-5A [25]. Other materials can be employed as well for the piezoelectric disk, such as PVDF copolymers [28] and piezoelectric single crystals [29]. The cap was made of brass with a density, Young’s modulus, and Poisson’s ratio of 7799 kg/m^3^, 90 GPa, and 0.35, respectively. The coating material was RTV-3460 (Elkem, Oslo, Norway), with a density of 1198 kg/m^3^ and a sound speed of 983 m/s. The dimensions of the cymbal transducer and array specimen were the same as those used in [10,27].

**Figure 11:**
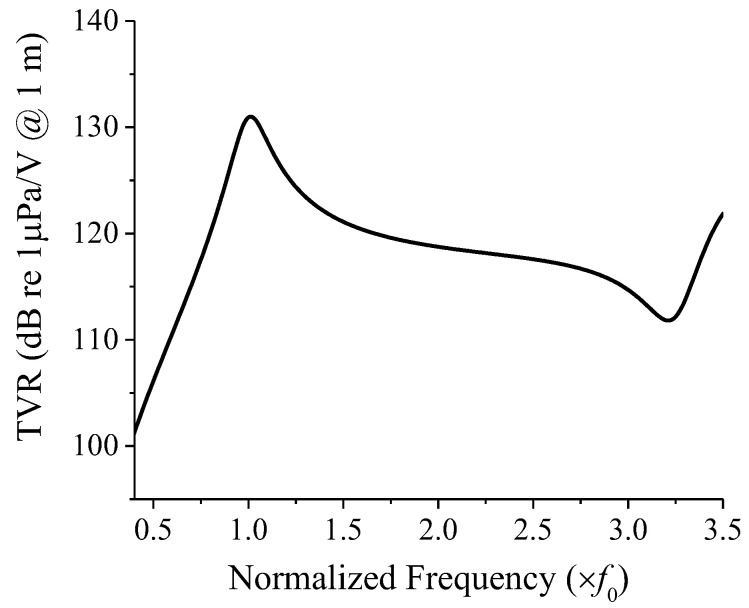
TVR spectrum of the immersed cymbal transducer calculated with the equivalent circuit in Figure 2.

Figure 11 shows the TVR spectrum of an immersed cymbal transducer calculated using the equivalent circuit in Figure 2 and the commercial software MATLAB^®^ 2021b (MathWorks^®^, Natick, MA, USA). The peak TVR frequency of the transducer was $f_{0}$, and the fractional bandwidth was 18.2%. The material and dimensions of the cymbal transducer used in this study were the same as those in [10]. The TVR spectrum calculated using equivalent circuit analysis (ECA) was verified through comparison with those from finite element analysis (FEA) and measurements in [10].

**Figure 12:**
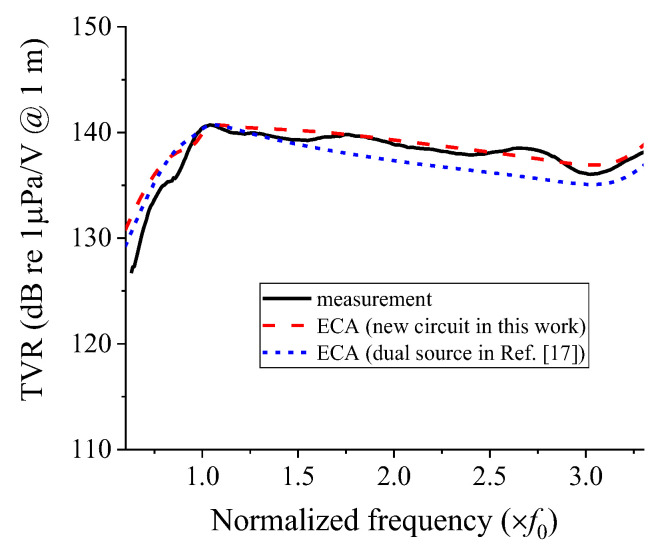
TVR spectra of the cymbal array from the measurement and ECA [17].

Figure 12 presents the TVR spectra of the array obtained from the measurement and analysis using the equivalent circuit in Figure 4. Figure 12 presents the results of two ECA cases: analysis using the new acoustic impedance network in Equation (10) developed in this work and that using the radiation impedance by considering a cymbal transducer as a dual-piston source, as suggested in [17]. The measurement of the TVR spectrum of the cymbal array specimen was conducted using the same facility and procedure described in [15]. A water tank of 5 m in length and 3 m in depth was used for the measurement, and the cymbal array specimen and a hydrophone (Hydrophone TC4033, Teledyne RESON, Denmark) were submerged to a depth of 1.5 m with a 1.5 m distance apart from each other. In the ECA, the impedance of the electrical system to drive the array was not considered; therefore, there might be a discrepancy in the quantitative value of the TVR level. Hence, the calculated peak TVR level was adjusted to have the same magnitude as the measured value. The quantitative characteristics of the array from the measurement and ECA are summarized in Table 1.

In Table 1, the −3 dB fractional bandwidth calculated using the new acoustic impedance network in Equation (10) and using the dual-piston source idea studied in [17] differed from the measured values by 2.4% and 26.2%, respectively. This shows that the acoustic impedance network developed in this work was more accurate in reflecting the interaction between the cymbal transducers in the array, which resulted in a good agreement of the analysis results with the measured data, and thus a more accurate analysis of the array performance.

## 4. Efficiency of the Equivalent Circuit Analysis

**Figure 13:**
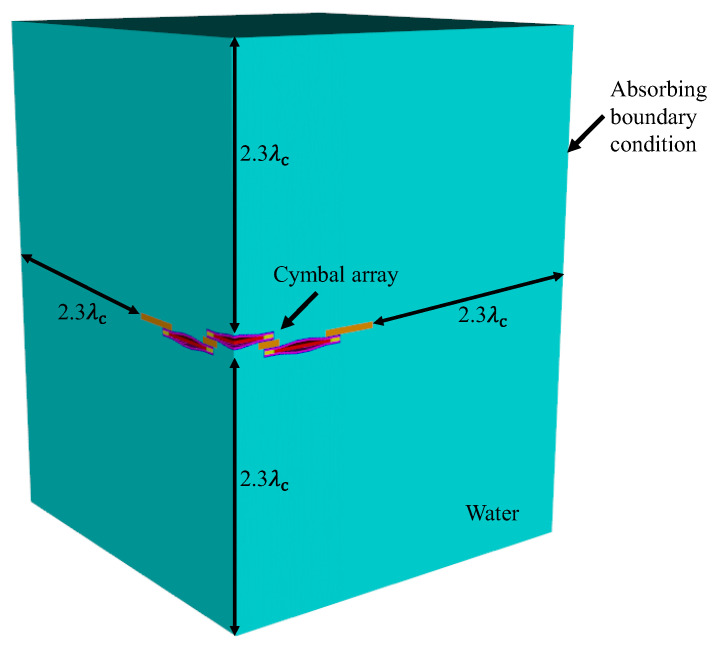
Finite element model of the immersed 3 × 3 cymbal array.

The main purpose of developing an equivalent circuit for the cymbal array is to increase the efficiency of the analysis. To verify the efficiency of the ECA developed in this study, the time required to execute the ECA for a cymbal array was compared with that of the FEA. A commercial software package, PZFlex^®^ (Weidlinger Associates, New York, NY, USA) was used for the FEA. Figure 13 shows the FEA model used to analyze the transmission characteristics of an immersed 3 × 3 cymbal array. A quarter model was used to reduce the analysis time by utilizing the symmetry of the array structure. The array was surrounded by sufficient water to preserve the far-field distance from the array to a measurement point around the edge. The distance from the edges of the array to the water boundary was 2.3 $\lambda_{c}$, where $\lambda_{c}$ is the wavelength at the center frequency of the array. All outer boundaries of the water were enforced with sound-absorbing conditions to prevent any reflection of waves at the boundary. The smallest element size in the FEA model was set to $\lambda_{c}$/400 to smoothly depict the curved surface of the cap, and the total number of elements constituting the model was 2.1 billion.

**Figure 14:**
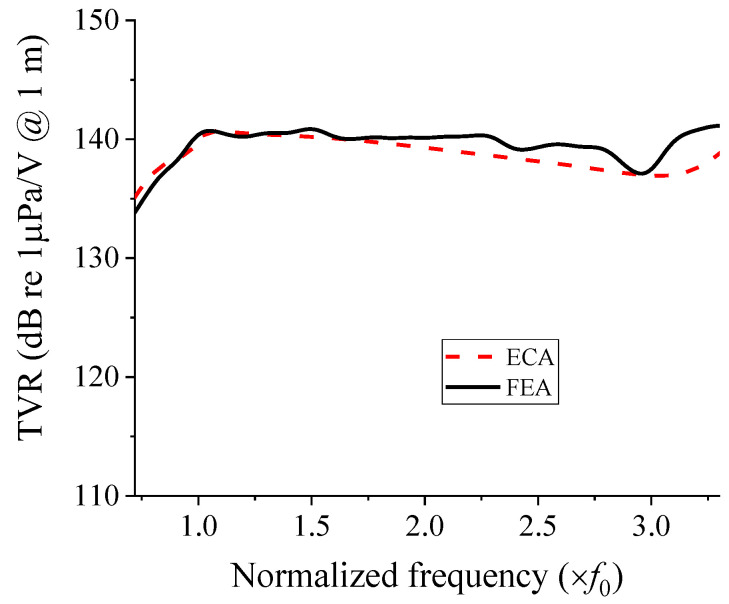
Comparison of the TVR spectra from the FEA and ECA.

Figure 14 shows the TVR spectrum obtained from the FEA of the cymbal array in comparison with that from the ECA using the acoustic impedance network developed in this study. The same computer with 64-bit Windows 10 was used for both the FEA and ECA, with the following specifications: Intel^®^I9-9940X CPU @ 3.30 GHz (Intel^®^, Santa Clara, CA, USA), and 128 GB RAM.

The spectrum from the ECA is the same as that obtained using the new circuit in Figure 12. In the TVR spectrum from the FEA, the peak TVR frequency and fractional bandwidths were 1.05 *f*_0_ and 105.9%, respectively. The discrepancy with the ECA results in Table 1 was less than 1.1% for both the peak TVR frequency and fractional bandwidth; thus, there is good agreement. 

The calculation time required to obtain the TVR spectra in Figure 14 was 28 s for the ECA and 3 d 6 h 2 min 32 s for the FEA; therefore, the calculation speed of the ECA was 10,034 times faster than that of the FEA. This result confirms that the equivalent circuit method had a superior calculation speed compared to the FEM. Therefore, the equivalent circuit developed in this study can lead to efficient analysis of the acoustic characteristics of the cymbal array without the time constraint encountered in the FEA.

## 5. Conclusions

Previous studies related to cymbal arrays used the FEM to analyze acoustic characteristics. The FEM usually requires a longer analysis time and large computation resources. Although the ECM is a more efficient method in this regard, an equivalent circuit exclusively for wideband cymbal arrays has not yet been available. In addition, the interaction between elements in the cymbal array was not accurately represented in the equivalent circuit of previous works [17].

In this study, we proposed a new equivalent circuit for analyzing the acoustic characteristics of a cymbal array. The proposed equivalent circuit includes a new acoustic impedance network that accurately reflects the interaction between the upper and lower caps of cymbal transducers in the array. The accuracy and validity of the newly developed equivalent circuit were verified by comparing the TVR spectrum of a cymbal array analyzed using the new circuit with that from the measurement. The difference between the bandwidth obtained from the measurement and the calculated results was merely 2.4%, and the new method was observed to be more accurate than the previously presented method. The efficiency of the new equivalent circuit was confirmed by comparing the analysis speed of the ECA with that of the FEA under the same calculation conditions, wherein the calculation speed of the ECA was 10,034 times faster than that of the FEA. 

Therefore, the new equivalent circuit can facilitate the analysis and design of large arrays of cymbal transducers with higher efficiency. Future work may include the application of the acoustic impedance network developed in this work to the analysis of other acoustic transducers with dual radiating surfaces, such as flextensional and bender transducers.

## Figures and Tables

**Table 1:** Measured and analyzed acoustic characteristics of the cymbal array.

Method	Peak TVR Frequency ($\times f_{0}$)	−3 dB Fractional Bandwidth (%)
Measurement	1.02	102.4
ECA with the new acoustic impedance network	1.06	104.8
ECA with the dual-source network	1.05	76.2

## Data Availability

Not applicable.

## References

[1] Dogan A., Fernandez J.F., Uchino K., Newnham R.E. The “Cymbal” Electromechanical Actuator. Proceedings of the 10th IEEE International Symposium on Applications of Ferroelectrics.

[2] Zhang J., Hughes W.J., Bouchilloux P., Meyer R.J., Uchino K., Newnham R.E. (1999). A class V flextensional transducer: The cymbal. Ultrasonics.

[3] Tressler J.F., Cao W., Uchino K., Newnham R.E. Ceramic-Metal Composite Transducers for Underwater Acoustic Applications. Proceedings of the 10th IEEE International Symposium on Applications of Ferroelectrics.

[4] Dogan A., Uzgur E. (2006). Size and material effects on cymbal transducer for actuator applications. Ferroelectrics.

[5] Zhang J., Hughes W.J., Hladky-Hennion A.C., Newnham R.E. (1999). Concave cymbal transducers. Mater. Res. Innov..

[6] Bejarano F., Feeney A., Lucas M. (2014). A cymbal transducer for power ultrasonics applications. Sens. Actuator A Phys..

[7] Choi Y., Shim H., Roh Y. (2019). Comparative analysis of the acoustic characteristics of different types of cymbal transducers. J. Acoust. Soc. Korea.

[8] Shim H., Roh Y. (2019). Design and fabrication of a wideband cymbal transducer for underwater sensor networks. Sensors.

[9] Shim H., Roh Y. (2021). Development of an equivalent circuit of a cymbal transducer. IEEE Sens. J..

[10] Shim H., Kim K., Seo H., Roh Y. (2022). New equivalent circuit of a cymbal transducer incorporating the third harmonic mode of vibration. IEEE Sens. J..

[11] Tressler J.F., Newnham R.E., Hughes W.J. (1999). Capped ceramic underwater sound projector: The “cymbal” transducer. J. Acoust. Soc. Am..

[12] Zhang J., Hladky-Hennion A.C., Hughes W.J., Newnham R.E. (2001). Modeling and underwater characterization of cymbal transducers and arrays. IEEE Trans. Ultrason. Ferroelectr. Freq. Control.

[13] Rajapan D., Rajeshwari P.M., Sankar M., Trinath K., Prasad N.S. Miniaturized Underwater Sensors for the Realization of Conformal Arrays. Proceedings of the Oceans 2006 Asian Pacific.

[14] Du Y.Q., Dai R., Pang D.P. (2011). Research on fabrication techniques and performance analysis for 3 × 3 cymbal transducer array. Adv. Mat. Res..

[15] Kim D., Shim H., Oh C., Kim K., Seo H., Roh Y. (2021). Design of a broadband array pattern of underwater cymbal transducers. Sensors.

[16] Pyo S., Roh Y. (2020). Structural design of an acoustic planar array transducer by using the equivalent circuit method. Ultrasonics.

[17] Zhang J., Hughes W.J., Meyer R.J., Uchino K., Newnham R.E. (2000). Cymbal array: A broad band sound projector. Ultrasonics.

[18] Lee H., Tak J., Moon W., Lim G. (2004). Effects of mutual impedance on the radiation characteristics of transducer arrays. J. Acoust. Soc. Am..

[19] Köymen H., Atalar A., Taşdelen A.S. (2017). Bilateral CMUT cells and arrays: Equivalent circuits, diffraction constants, and substrate impedance. IEEE Trans. Ultrason. Ferroelectr. Freq. Control.

[20] Xu T., Zhao L., Jiang Z., Guo S., Li Z., Yang P., Luo G., Sun L., Zhang L. (2020). Equivalent circuit models of cell and array for resonant cavity-based piezoelectric micromachined ultrasonic transducer. IEEE Trans. Ultrason. Ferroelectr. Freq. Control.

[21] Pyo S., Lim Y., Roh Y. (2021). Analysis of the transmitting characteristics of an acoustic conformal array of multimode tonpilz transducers by the equivalent circuit method. Sens. Actuator A Phys..

[22] Oguz H.K., Atalar A., Köymen H. (2013). Equivalent circuit-based analysis of CMUT cell dynamics in arrays. IEEE Trans. Ultrason. Ferroelectr. Freq. Control.

[23] Akhbari S., Sammoura F., Lin L. (2016). Equivalent circuit models for large arrays of curved and flat piezoelectric micromachined ultrasonic transducers. IEEE Trans. Ultrason. Ferroelectr. Freq. Control.

[24] Sim M.J., Jeong W.B., Hong C. (2021). An equivalent circuit based electro-vibro-acoustic model of a cylindrical transducer array. J. Acoust. Soc. Am..

[25] Butler J.L., Sherman C.H. (2016). Transducers and Arrays for Underwater Sound.

[26] Xu T., Zhao L., Jiang Z., Guo S., Li Z., Yang P., Luo G., Sun L., Zhang L. (2021). Equivalent circuit model for a large array of coupled piezoelectric micromachined ultrasonic transducers with high emission performance. IEEE Trans. Ultrason. Ferroelectr. Freq. Control.

[27] Mudiyala J., Shim H., Kim D., Roh Y. (2022). Development of a dual-layer structure for cymbal transducer arrays to achieve a wider bandwidth. Sensors.

[28] Ahmed A., Jia Y., Huang Y., Khoso N.A., Deb H., Fan Q., Shao J. (2019). Preparation of PVDF-TrFE based electrospun nanofibers decorated with PEDOT-CNT/rGO composites for piezo-electric pressure sensor. J. Mater. Sci. Mater. Electron..

[29] Zhang S., Li F., Luo J., Sahul R., Shrout T.R. (2013). Relaxor-PbTiO3 Single Crystals for Various Applications. IEEE Trans. Ultrason. Ferroelectr. Freq. Control.

